# MiR-126 Suppresses the Glucose-Stimulated Proliferation via IRS-2 in INS-1 β Cells

**DOI:** 10.1371/journal.pone.0149954

**Published:** 2016-02-26

**Authors:** Hong Tao, Meng-meng Wang, Man Zhang, Shao-ping Zhang, Chun-hui Wang, Wen-jun Yuan, Tao Sun, Lan-jie He, Qi-kuan Hu

**Affiliations:** 1 The Department of Physiology, Ningxia Medical University, Yinchuan, China; 2 Jiamusi Central Hospital, Department of Epilepsy, Jiamusi, Hei Long Jiang, China; 3 Ningxia Key Lab of Cerebrocranial Diseases, the National Key Laboratory Incubation Base, Yinchuan, China; 4 General Hospital of Ningxia Medical University, Yinchuan, China; 5 The Department of Physiology, Second Military Medical University, Shanghai, China; The University of Hong Kong, CHINA

## Abstract

**Background:**

Increasing evidence suggests that miR-126 participates in the glucose homeostasis through its target molecules. Although bioinformatics analysis predicts that miR-126 can bind with the insulin receptor substrate-2(IRS-2) mRNA at the “seed sequence”, but there are still no definitely reports to support it. In this study, we provided evidences that IRS-2 was one of the target genes of miR-126. And miR-126 has a proliferation inhibiting effects in INS-1 β cells, mainly through the suppression of IRS-2.

**Methods:**

The 3’-UTR of IRS-2 regulated by miR-126 was analyzed by the luciferase assay and western blot. Furthermore, proliferation of INS-1 β cells stimulated by glucose was tested, and the association between IRS-2 and miR-126 were analyzed.

**Results:**

We found that mutation of only three of the 6 “seed sequences” can eliminate the inhibition effect of miR-126. In INS-1 β cells, administration of miR-126 suppresses the proliferation, together with the unbalanced down-regulation of IRS-2 and IRS-1. Over-expression of IRS-2 can reverse the proliferation effect of miR-126, while not of IRS-1. These results suggested that miR-126 inhibited the β-cell proliferation via the inhibition of IRS-2 instead of IRS-1.Additionally, we also found that high glucose and insulin could stimulate the rapid production of endogenous miR-126 within 6 hours, together with the short term suppression of IRS-1 and IRS-2 expression, and intensify the unbalanced expression of IRS-1 and IRS-2.

**Conclusions:**

IRS-2 was one of the targets of miR-126. MiR-126 inhibited the β-cell proliferation through IRS-2 instead of IRS-1. MiR-126 may take part in the glucose homeostasis both through its target IRS-2 and IRS-1. The unbalance between IRS-1 and IRS-2 caused by miR-126 may play an important role in type 2 diabetes.

## Introduction

Several recent reports have correlated miR-126 with the insulin resistance and diabetes [1]. Studies in patients with type 2 diabetes find that their plasma miR-126 level is significantly lower in comparison with normal individuals, and the decreased miR-126 level is inversely associated with the development of diabetes [2]. One of the most important target genes of miR-126 is the insulin receptor substrate-1 (IRS-1) [3]. Some changes were linked to the down-regulation of IRS-1 by ectopic miR-126, such as the affected mitochondrial energy metabolism, reduced mitochondrial respiration, and promoted glycolysis, reducing Akt signaling, inhibiting cytosolic sequestration of FoxO1, promoted expression of genes involved in gluconeogenesis and oxidative stress defense in H28 cells [4].

Insulin receptor substrates 1 and 2 (IRS-1 and IRS-2) are the major insulin-signaling components regulating β-cell metabolism and survival. IRS-1 and IRS-2 depend on the same downstream IRS/PI3-kinase/Akt signaling cascade that is required for insulin regulation of glucose homeostasis [5]. These two receptors are functionally related closely together, and have very refined differences in the regulation of glucose homeostasis. It is suggested that IRS-1 plays a predominant role in somatic growth, while IRS-2 in glucose homeostasis, especially in liver [6]. Loss of IRS-2 causes diabetes in mice due to β-cell insufficiency and peripheral insulin resistance [7]. But loss of IRS-1 displays profound growth retardation without developing diabetes, perhaps because of the insulin secretion compensation for the presence of mild insulin resistance [8].

Interestingly, bioinformatics analysis also predicts that miR-126 can match with the “seed sequences” of IRS-2 mRNA. But there are still no reports to confirm it. Another hint correlating the IRS-2 and miR-126 is the proliferation inhibiting effects of miR-126 on β-cells. Over-expression of IRS-2 in isolated pancreatic islets causes proliferation and protects human β-cells from hyperglycemia-induced apoptosis [9]. This implies that miR-126 executes its β-cell proliferation inhibiting effects via IRS-2.Therefore; we design the experiments to test this hypothesis using the rat insulinoma (INS-1) β-cell line. We also try to explore the different physiological significance of the endogenous miR-126 in the glucose homeostasis through targeting the two insulin receptors, IRS-1 and IRS-2.

## Materials and Methods

### INS-1 β cells culture

The rat insulinoma (INS-1) β-cell line (AiYan Biotechnology Corporation, Ltd, Shanghai, China) was maintained in RPMI1640 medium (Gibco Corporation), supplemented with 10% fetal bovine serum, 50μm β-mercaptoethanol, 1mM sodium pyruvate (Gibco Corporation), 2mM L-Glutamine(Amresco), 5.6mM Glucose and 100 U/mL penicillin and streptomycin (Chinese Academy of Medical Sciences, Beijing, China) in 5% CO_2_ incubator at 37°C.

### MiRNA transfection

MiRNAs were transfected using Lipofectamine 2000 (Transgene, China) as directed. The following miRNAs were used: (1) miR-126 mimic(126M), a duplex RNA, with a sense (5’-UCGUACCGUGAGUA AUAAUGCG-3’), and an antisense sequence (5’-CAUUAUUACUCAC GGUACGAUU-3’); (2) miR-126 inhibitor(126I), with a sequence of 5’- CGCAUUAUUACUCACGGUACGA-3’; and (3) non-targeting negative control (NC), with a sense:(5’-UUCUCCGAACGUGUCACGUTT-3’), and antisense:(5’-ACGUGACACGUUCGGAGAAT-3’).

### Luciferase reporter plasmid construction and luciferase activity assay

The dual luciferase psiCheck2 reporter plasmid was used to generate the reporter containing the cloned IRS-2 3’-UTR fragment, which was amplified from human genomic DNA by PCR with the following primers: 5’-tccgcTCGAGAGCTGGAAGGTCAATTTCAGTG-3’ (sense) and 5’-atttgcGGCCGCACTCTACGGATAGAGGGCGAGT-3’ (antisense). The cloned fragment was inserted into psiCheck2 reporter plasmid via XhoI and NotI sites. The mutant reporter plasmid was screened by using the DpnI mediated site-directed mutagenesis method with the following primers: 5’-TGTAACTCCCCCCAGGCGTGATAGGGACTGAAT-3’ (sense) and 5’-ATTCAGTCCCTATCACGCCTGGGGGGAGTTACA-3’ (antisense).All plasmids were verified by direct sequencing.

For the luciferase assay, the INS-1 β cells were seeded into 24-well plates at 5×10^5^ densities. MiRNAs (40nM/well) and the psi-check2-IRS-2 reporter plasmids (40nM/well) were co-transfected. After 24 hours, cells were harvested and the luciferase activity was detected using the Dual Luciferase Reporter Assay System (Transgene, Beijing, China) as directed.

### RNA extraction and Real-time PCR

Total RNA was isolated with TRIzol reagent and reverse transcripted using Transcriptor First Strand cDNA Synthesis Kit. Real-time PCR was performed with SYBR Green PCR reagent (Transgene, Beijing, China) on a fluromax-4 Spectrofluorometer (Bio-Red). The fold changes in target mRNA expression were calculated by using the 2^-ΔΔCt^ method, normalized to GAPDH. The Q-PCR of miR-126 was normalized to the U6.

Primers were as follows: miR-126: 5’-GCTAGCTCGTACCGTTAA TAA-3’(sense) and 5’-ATTCTAGAGGCCGAGGCGGCCGACATGT-3’ (antisense); U6: 5’-CTCGCTTCGGCAGCACA-3’(sense) and 5’-AACG CTTCACGAATTTGCGT-3’(antisense);IRS-1:5’-AAATCCTCTTCTAACTCATGGGTACC-3’ (sense) and 5’-CAGTTTCAGCAGCAGATGAA ATGTA-3’ (antisense); IRS-2: 5’-TCGAGAGCTGGAAGGTCAATTTC AGTG-3’(sense) and 5’-GGCCGCACTCTACGGATAGAGGGCGAGT- 3’ (antisense); GAPDH:5’-GCACCGTCAAGGCTGAGAAC-3’(sense) and 5’-TGGTGAAGACGCCAGTGGA-3’ (antisense). All primers were custom-synthesized by Sangon Biotech (Shanghai) Co. Ltd, China.

### Western blot, Cell proliferation, Statistical analysis

INS-1 β cells were harvested and lysed to perform western blot routinely. 80μg of total protein were electrophoresed on a 10% (w/v) SDS-PAGE and transferred into PVDF membranes (0.45 mm, Millipore, Darmstadt, Germany). Antibodies used were as follows: rabbit anti-IRS-1, anti-IRS-2 (both from Immuno Way; both diluted 1:200); rabbit anti-GAPDH (ZhongShan, Beijing, China, 1:1000); HRP-conjugated secondary antibody (diluted 1:5000).

Cell proliferation was tested by the Cell Counting Kit-8 (CCK-8, Transgene, Beijing, China) according to the manufacturer’s instructions. Cells were first transfected with the miRNAs or plasmids 24 hours in 6-well plates and then reseeded into 96-well plates for testing. 3×10^3^ cells per well were seeded and the cell proliferation was detected 48 hours after transfection.

Statistical analyses were performed with GraphPad Prism 5.0 software. Two-tailed P < 0.05 was considered to be statistically significant.

## Results

### IRS-2 is a target gene of miR-126 in INS-1 β cells

**Fig 1:**
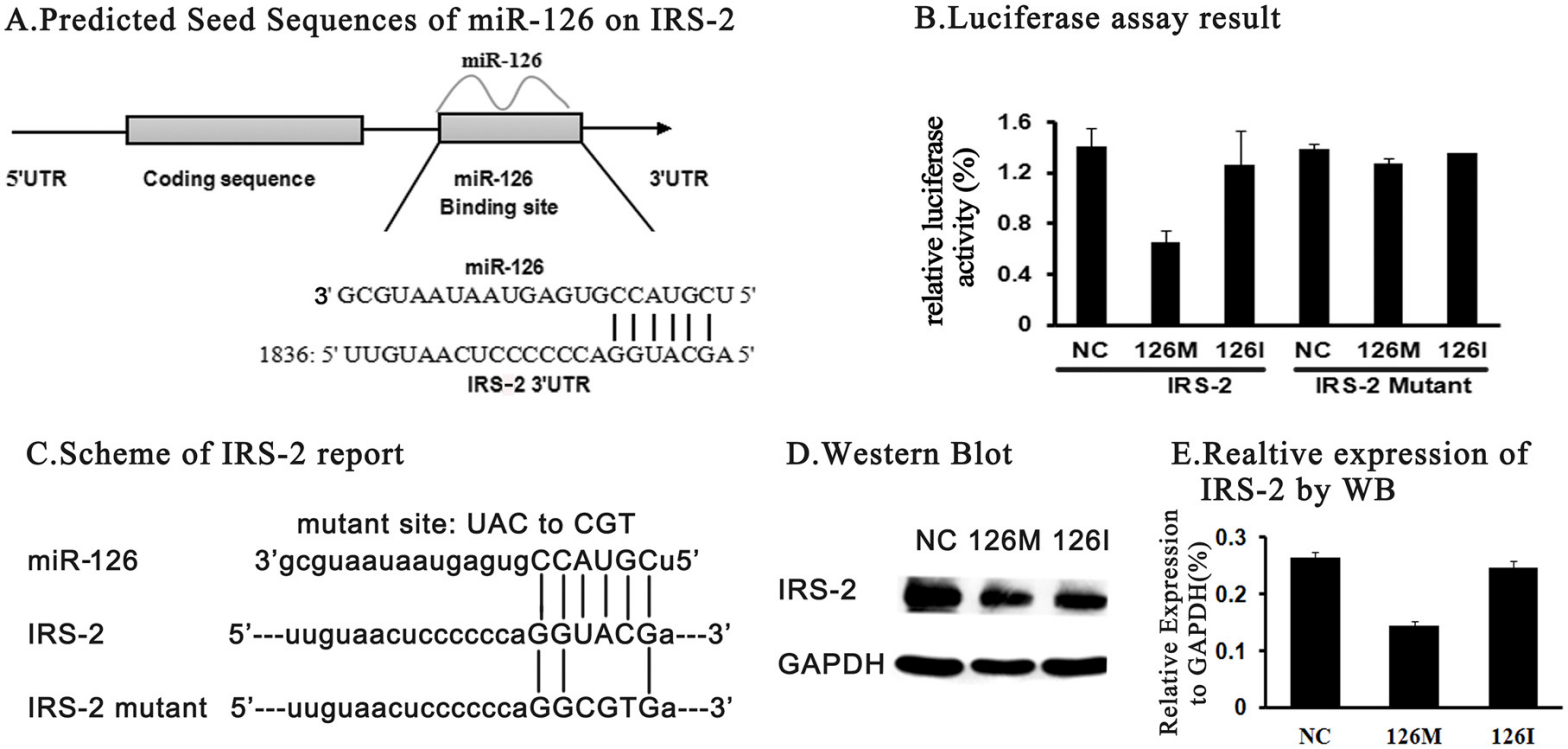
IRS-2 is a potential target of miR-126 in INS-1 β cells. (A)Bioinformatics analysis indicated that there was a 6 consecutive nucleotide seed sequences as the potential miR-126 binding site in the 3’ -UTR region of IRS-2 mRNA, as indicated with short vertical lines. (B) A 199bp fragment of IRS-2 3’-UTR region was cloned into psicheck-2 reporter plasmid. Then the luciferase reporter assay was performed by co-transfection of the luciferase reporter vector and the miR-126 mimic (126M), with the nonspecific sequence (NC) as the negative control. A complementary miR-126 inhibitor (126I) was designed to specifically block the miR-126, both endogenously and exogenously. Then the luciferase reporter assay was re-performed with the mutant reporter vector, co-transfected with the nonspecific sequence (NC), miR-126 mimic (126M), miR-126 inhibitor (126I) respectively. Luciferase activity was tested 24 h after transfection. (C) The IRS-2 -psicheck-2 reporter plasmid was mutated at the center of the miR-126 seed sequences. (D, E) The expression of the IRS-2 protein was detected by western blot. The relative levels were normalized with the GAPDH and represented as a column chart.

Bioinformation from the mirBase web showed that IRS-2 was the potential target of miR-126-3p. The predicted seed sequences contained 6 consecutive nucleotides CCAUGC at the 3’-UTR region of IRS-2 mRNA (Fig 1A). In order to test if it was true, a series of experiments was designed as follows.

First, we cloned a 199bp fragment from the 3’-UTR of IRS-2 mRNA by PCR. Then we inserted this fragment into the psicheck-2 reporter plasmid for luciferase assay. As predicted, the 126M could inhibit the reporter activity significantly(about 50%), which indicated that the 126M bound to the 3’-UTR of IRS-2 and blocked the transcription of the IRS-2 promoted reporter transcription(Fig 1B). Second, to further proof the specific binding of miR-126 at the predicted seed sequences, a mutant reporter plasmid with 3 nucleotides was constructed at the center of the 6 seed sequences (Fig 1C). As expected, the mutant reporter had no response to the 126M transfection, as shown in Fig 1B. This result demonstrated that the three nucleotides UAC was crucial for the binding of miR-126 with the IRS-2.

Third, the protein levels of IRS-2 expression were determined by western blot. As shown in Fig 1D and 1E, 126M transfection decreased markedly the expression of the IRS-2 strongly. This inhibition effect of 126M could be reversed almost entirely by the 126I. IRS-2 proteins rescued the control level after transfected with the 126I.

In general, the above results indicated that IRS-2 was one of the target genes of miR-126.

### MiR-126 affects the proliferation of INS-1 β cells through the inhibition of IRS-2

**Fig 2:**
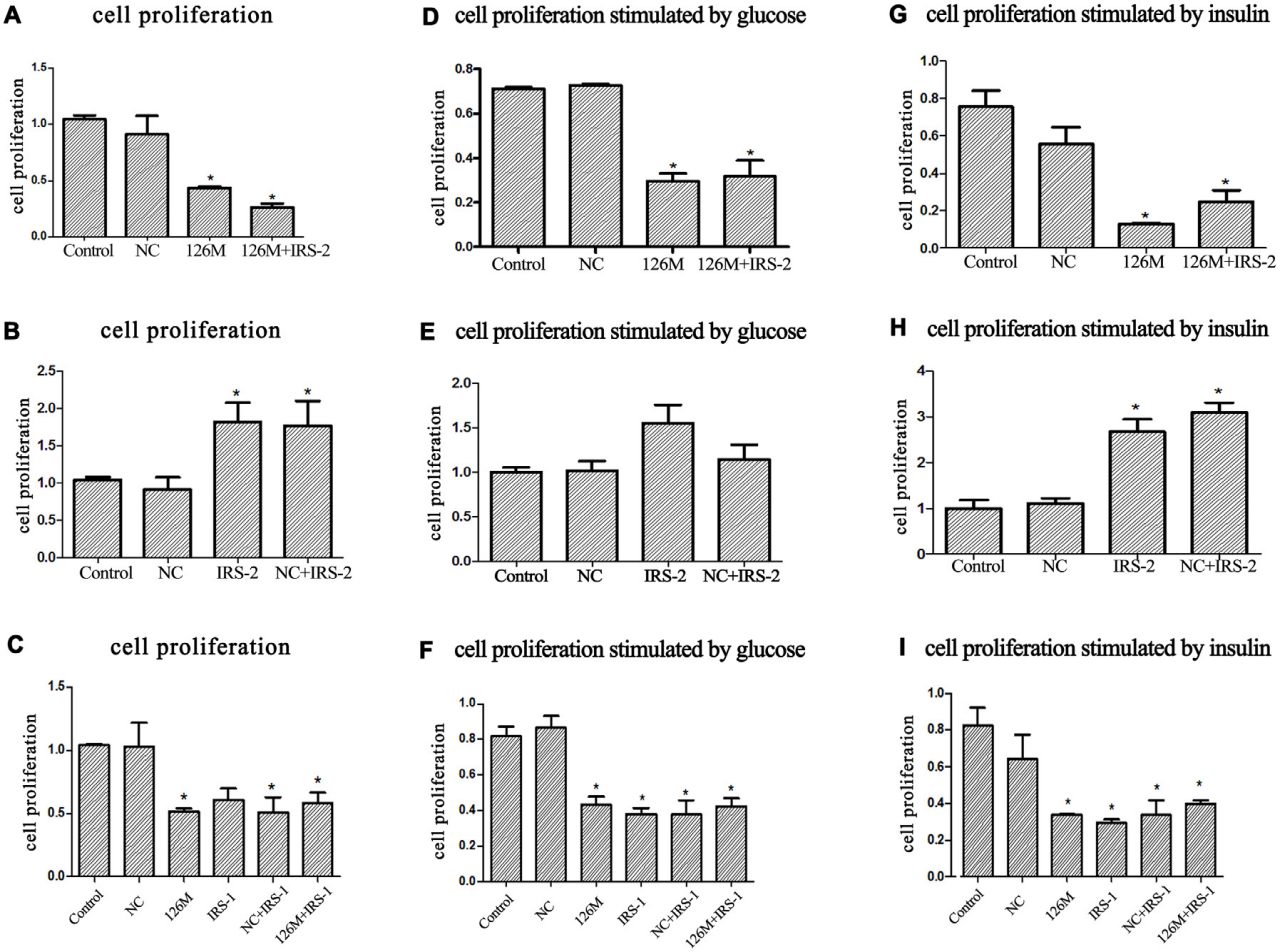
INS-1 β cells proliferation after transfection. (A,B,C)Cells were cultured in normal glucose medium (11.1mM) and treated as indicated. (D,E,F)Cells were cultured in high glucose (22.2mM). (G,H,I) Cells were cultured in medium containing insulin. The proliferation rates were detected by CCK-8 method after transfected for 48 hours.*: vs NC p<0.05

It is reported that the major function of IRS-2 in β cells is to regulate the proliferation. Loss of IRS-2 causes β-cell dysfunction characterized by a 50% reduction in β-cell mass, with diabetic phenotypes [4]. Therefore, the possible function of miR-126 binding with the IRS-2 is the regulation of β-cell proliferation. To test this, the proliferation of INS-1 β cells was investigated by CCK-8 methods. As shown in Fig 2A and 2B, the transfection of 126M has a strong inhibitive effect on the cell proliferation. On the other hand, the transfection of IRS-2 expressive vector stimulates the cell proliferation as expected.

To determine if the proliferation inhibition effect of miR-126 was through the specifically binding to the IRS-2, we further investigated the combination of miR-126 and the IRS-2 co-transfection effect. The cell proliferation was still inhibited by the miR-126 plus the IRS-2 co-transfection, compared both with the NC plus IRS-2 control and naive control. These results suggested that miR-126 suppressed the proliferation mainly through the inhibition of IRS-2.

As a contrast, the effect of IRS-1 was tested simultaneously. IRS-1 was mainly responsible for the growth and had a little effect on the proliferation of INS-1 β cells [4]. In our experiments, transfection of IRS-1 expressive vector not only did not stimulate the proliferation, but also even inhibited cell proliferation, in contrast to the increasing effect of IRS-2. The combination of miR-126 and the IRS-1 co-transfection did not show any significant changes on the cell proliferation, compared with the two control groups, the IRS-1 and the NC plus IRS-1 (Fig 2C).

Considering that the physiological responses through IRS-2 were often combined with the stimulation of glucose and insulin, so we further tested the proliferation effects under the stimulation of high glucose and insulin. Under the continuous stimulation of high glucose (22mM), miR-126 behaved as an inhibitor on the cell proliferation, while IRS-2 behaved as the proliferation stimulator, as the contrast. The combination of miR-126 and the IRS-2 co-transfection displayed the similar inhibition effect on cell proliferation (Fig 2D and 2E). IRS-1 still showed an inhibitive effect on cell proliferation (Fig 2F). The combination of miR-126 and the IRS-1 co-transfection did not show any significant changes on the cell proliferation, when compared with the two control groups, the IRS-1 and the NC+IRS-1 (Fig 2F).

As for the continuous stimulation of insulin, miR-126 had a stronger inhibition effect on the cell proliferation, while IRS-2 increased the cell proliferation strongly. The miR-126 and the IRS-2 co-transfection still inhibited cell proliferation significantly (Fig 2G and 2H). On the other hand, the tendency of IRS-1 still behaved the same, too. IRS-1 inhibited the cell proliferation dramatically, still with the similar proliferation rate in the miR-126+IRS-1 group (Fig 2I).

### High glucose stimulates the endogenous miR-126 production and affects both the IRS-2 and IRS-1 expression

**Fig 3:**
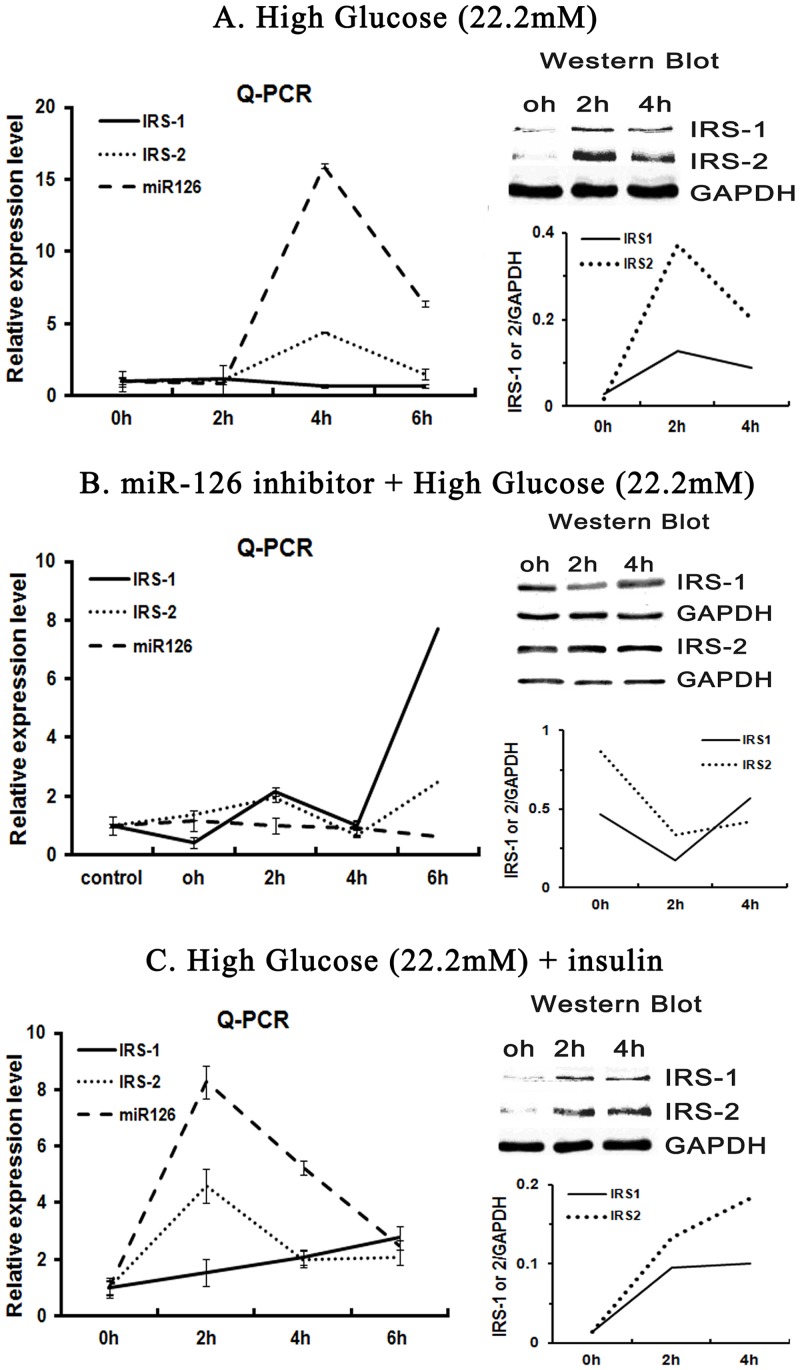
High glucose and insulin stimulates the endogenous expression of miR-126. The endogenous expression of miR-126, IRS-1 and IRS-2 levels were detected by Q-PCR and western blot. High glucose and insulin was administrated into the culture medium respectively. Cells were harvested at the indicated time. (A) High glucose stimulated a fast increasing wave of endogenous miR-126 expression within 6 hours in INS-1 β cells. Both the transcription and protein expression of IRS-1 and IRS-2 showed an increase together with the miR-126 wave. (B) INS-1 β cells were transfected with miR-126 inhibitor for 24h. Then these cells were stimulated by high glucose and were harvested at the indicated time. The expression of the endogenous miR-126 was inhibited expectedly. The transcription and protein expression of both IRS-1 and IRS-2 kept on a low level during 6 hours. But the IRS-1 mRNA expression increased dramatically after 6 hours. (C) INS-1 β cells stimulated by high glucose and insulin behaved similarly with the high glucose alone. The endogenous miR-126 wave still appeared but the peak of it came earlier, at about 2 hours. Both the transcription and protein expression of IRS-1 and IRS-2 showed an increase together with the miR-126 wave again.

High glucose and insulin are the two major feedback stimuli in the β-cell behavior. In hepatic cells, the PI3K activity associated with IRS-2 began to increase during fasting, reached its peak immediately after re-feeding, and decreased rapidly thereafter [6]. This suggests that IRS-2 responds to the high glucose stimulus in a rapid mode. Whether the IRS-2 expression responds to the high glucose in the β-cell, or whether the endogenous miR-126 could be produced during the high glucose stimulus, is still unknown. We explored these problems by detecting the miR-126 expression using Q-PCR. For the expectation that miR-126 and IRS-2 may respond to the high glucose very rapidly, we designed to harvest cells during the early 2 hours, 4 hours, and 6 hours respectively. The expression of the endogenous miR-126 showed a very rapid response to the high glucose stimulus, reached its peak at about 4 hours, and then came down to the normal level. This response can be counteracted by the specific 126I (Fig 3A and 3B). But miR-126 and IRS-2 reached their peak at about 2 hours when they were stimulated by high glucose and insulin at the same time (Fig 3C).

Together with the up-regulation wave of the endogenous miR-126, the expression of IRS-2 and IRS-1 mRNA displayed a different mode of changes. The IRS-2 increased to a little peak while the IRS-1 even decreased. But the protein levels of IRS-2 and IRS-1 both increased (Fig 3A–3C), which suggested that IRS-1 and IRS-2 had other unknown regulators during this period.

## Discussions

Increasing evidence suggests that miR-126 participates in the glucose homeostasis through its target molecules. Recent studies identified circulating miR-126 as a biomarker for myocardial injury and vascular damage in diabetes. In a study including 160 patients, serum miR-126 was significantly lower in IGT/IFG (impaired glucose tolerance/impaired fasting glucose) subjects. And insulin plus diet control and exercise treatment could re-upregulate the serum miR-126 significantly [2]. In miR-126 knockdown mouse, the levels of peripheral blood glucose increased significantly with pathological abnormality in pancreas and liver [10, 11].

Several studies reported that miR-126 took the IRS-1 as its target gene and interfering with the mitochondrial function [3]. MiR-126 was up-regulated by oxidative stress in nonmalignant mesothelial (Met5A) and MM (H28) cell lines. The IRS-1-modulated ATP-citrate lyase deregulations were linked to the down-regulation of IRS-1 by ectopic miR-126, reducing Akt signaling and inhibiting cytosolic sequestration of FoxO1, which promoted the expression of genes involved in gluconeogenesis and oxidative stress defense [3]. Endothelial miR-126 repressed FoxO3, B-cell lymphoma 2, and IRS-1 mRNAs in the co-cultured SMCs [12]. However, there was still no evidence to elucidate the expression of miR-126 and its function with IRS-1 in the β cells. In the current study, we have first validated the suppression of IRS-1 by miR-126 at the mRNA and protein level, especially in the INS-1 β cell line.

Furthermore, we tested if the other similar molecule, IRS-2, could be suppressed by the miR-126. Although IRS-1 and IRS-2 depend on the same downstream IRS/PI3-kinase/Akt signaling cascades [5, 6], the refined differences between these two molecules have been reported. Ablation of the IRS-2 gene produced a diabetic phenotype; Mice lacking IRS-2 displayed peripheral insulin resistance and β-cell dysfunction characterized by a 50% reduction in β-cell mass. In contrast, the deletion of IRS-1 retarded somatic growth and enhances β-cell mass [6].These results suggested that IRS-2 predominantly mediated the proliferation of β-cells. In our experiments, miR-126 not only suppressed the expression of IRS-2, but also inhibited cell proliferation strongly in the INS-1 β cells. Over-expression of IRS-2 rescued the proliferation inhibitory effect of miR-126. In contrast, over-expression of IRS-2 and miR-126 could not rescue the proliferation inhibitory effect of miR-126.

**Fig 4:**
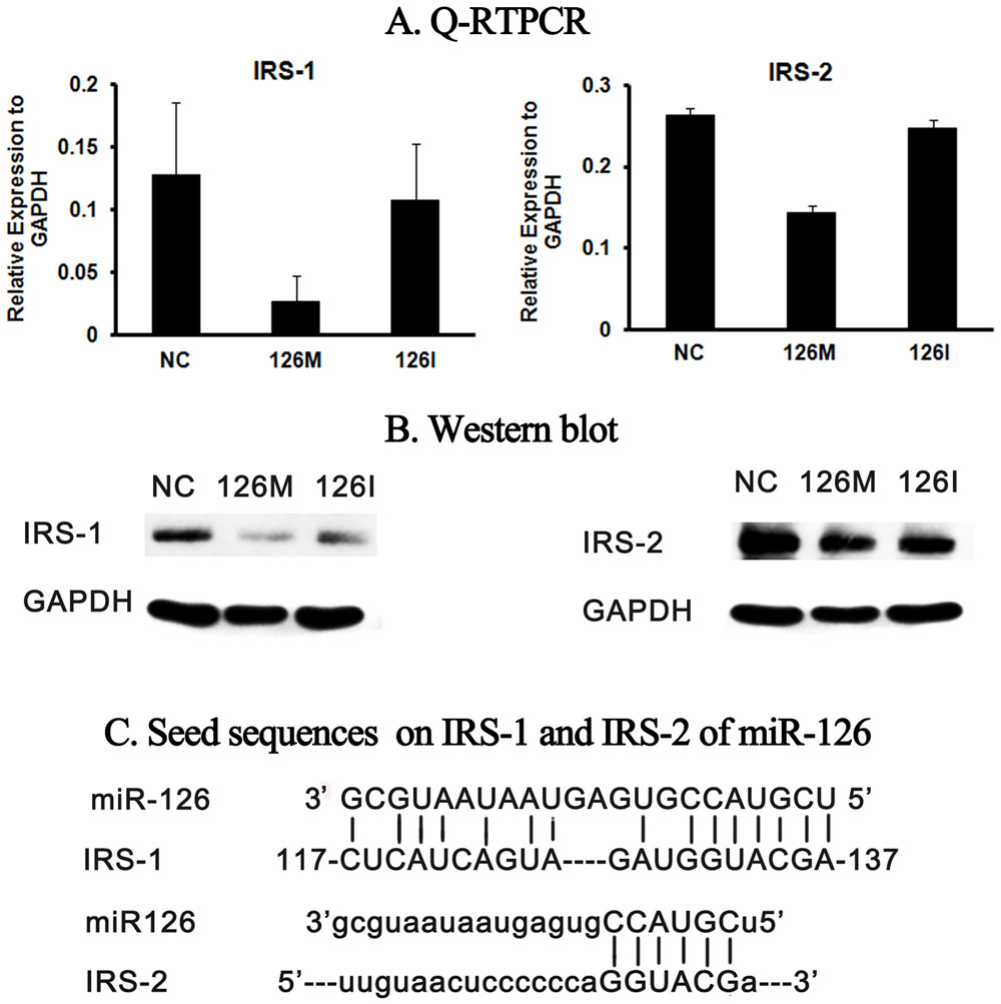
MiR-126 suppresses IRS-1 stronger than IRS-2. INS-1 β cells were transfected with miRNAs for 24 hours and then were harvested for Q-RTPCR and western blot. (A) Q-RTPCR results. (B) Western blot results. (C) Compare of the seed sequences binding by miR126 on the IRS-1 and IRS-2 3’-UTR region. Nonspecific sequence (NC), miR-126 mimic (126M), miR-126 inhibitor (126I).

Physiologically, the expression and the function of IRS-1 and IRS-2 should keep fine balance to maintain the homeostasis of the β cells. IRS-1 is responsible for the growth while IRS-2 is responsible for the proliferation of β cells. MiR-126 targeted both IRS-1 and IRS-2, but the strength of suppression on these two molecules was very different. Both Q-RTPCR and western blot experiments demonstrated that the inhibition effect of miR-126 on IRS-1 was stronger than that on the IRS-2 (Fig 4A and 4B). It is possible that there are more miR-126 binding sites on IRS-1 than that on IRS-2 (Fig 4C).

Therefore, administration of miR-126 may disturb the balance between IRS-1 and IRS-2. One possible result of the lower miR-126 was that the between IRS-1 and IRS-2 unbalanced, and the expression and function of IRS-1 were more distinguishable. In type 2 diabetes, serum miR-126 was significantly lower in IGT/IFG subjects [2], which support our prediction.

In hepatic cells, the PI3K activity associated with IRS-2 began to increase during fasting, reached its peak immediately after refeeding, and decreased rapidly thereafter. By contrast, the PI3K activity associated with IRS-1 began to increase a few hours after refeeding and reached its peak thereafter. The data indicate that IRS-2 mainly functions during fasting and immediately after refeeding, while IRS-1 functions primarily later after refeeding[13, 14].

Our experiment demonstrated that high-glucose stimulation (mimic the refeeding) caused a rapid (about 4h) up-regulation of miR-126 in INS-1 β cells. Consistently, the increasing peaks of IRS-1 and IRS-2 were suppressed during this time. The IRS-1 reached its peak later (6h). This result was consistent with the later effect of IRS-1 during refeeding. The fast up-regulation peak of endogenous miR-126 in INS-1 β cells implied the physiological role of miR-126 during the fasting and feeding. High glucose peak may cause a rapid production of miR-126 and change the balance between IRS-1 and IRS-2.

In patients with type 2 diabetes mellitus, serum miR-126 was significantly lower in IGT/IFG subjects. In the study of IRS-1 and IRS-2 knockout mice, the balance between IRS-1 and IRS-2 was demonstrated a crucial role during the insulin-tolerance test (ITT) conducted after fasting [13]. In the IRS-1 knockout mice, oral glucose-tolerance test (OGTT) conducted after fasting could not cause the blood glucose and serum insulin levels up regulated [14]. On the other hand, in the IRS-2-knockout mice the blood glucose levels after glucose loading were significantly elevated [10, 11]. Considering these two studies and the results of our study, we propose that miR-126 may play a crucial role in the regulation of glucose response during fasting and refeeding via its targeting suppression on the IRS-1 and IRS-2. The lower of serum miR-126 in type 2 diabetic patients may cause the unbalance of IRS-1 and IRS-2 and therefore promote the pathological injury of β cells.

## Conclusions

IRS-2 was one of the targets of miR-126. MiR-126 inhibited the β-cell proliferation through IRS-2 instead of IRS-1. MiR-126 may take part in the glucose homeostasis both through its target IRS-2 and IRS-1. The unbalance between IRS-1 and IRS-2 caused by miR-126 may play an important role in type 2 diabetes.

## References

[1] Meister J, Schmidt MH. MiR-126 and miR-126*: new players in cancer. Scientific World Journal 2010; 10:2090–100. 10.1100/tsw.2010.198 20953557 PMC5763667

[2] Zhang T, Lv C, Li L, Chen S, Liu S, Wang C, et al Plasma miR-126 is a potential biomarker for early prediction of type 2 diabetes mellitus in susceptible individuals. Biomed Res Int 2013; e761617 10.1155/2013/761617 PMC3886569 24455723

[3] Li H, Meng F, Ma J, Yu Y, Hua X, Qin J, et al Insulin receptor substrate-1 and Golgi phosphoprotein 3 are downstream targets of miR-126 in esophageal squamous cell carcinoma. Oncol Rep 2014; 32:1225–33. 10.3892/or.2014.3327 25017784

[4] Ryu HS, Park SY, Ma D, Zhang J, Lee W.The induction of microRNA targeting IRS-1 is involved in the development of insulin resistance under conditions of mitochondrial dysfunction in hepatocytes. PLoS One 2011;6: e17343 10.1371/journal.pone.0017343 21464990 PMC3064581

[5] Zhu N, Zhang D, Xie H, Zhou Z, Chen H, Hu T, et al Endothelial-specific intron-derived miR-126 is down-regulated in human breast cancer and targets both VEGFA and PIK3R2. Mol Cell Biochem 2011; 351:157–64. 10.1007/s11010-011-0723-7 21249429

[6] Burks DJ, White MF.IRS proteins and beta-cell function. Diabetes 2001;50 Suppl 1:S140–5. 11272176 10.2337/diabetes.50.2007.s140

[7] Araki E, Lipes MA, Patti ME, Brüning JC, Haaq B 3rd, Johnson RS, et al Alternative pathway of insulin signaling in mice with targeted disruption of the IRS-1 gene. Nature 1994 372:186–190. 7526222 10.1038/372186a0

[8] Withers DJ, Gutierrez JS, Towery H, Burks DJ, Ren JM, Previs S, et al Disruption of IRS-2 causes type 2 diabetes in mice. Nature 1998; 391:900–904. 9495343 10.1038/36116

[9] Mohanty S, Spinas GA, Maedler K, Zuellig RA, Lehmann R, Donath MY, et al Overexpression of IRS-2 in isolated pancreatic islets causes proliferation and protects human beta-cells from hyperglycemia-induced apoptosis. Exp Cell Res 2005; 303:68–78. 15572028 10.1016/j.yexcr.2004.09.011

[10] Hu Y, Li Y, Chen C, Zhu S, Guo M, Liu S, et al Identification of miR-126 knockdown mouse and the change of blood glucose. Zhong Nan Da Xue Xue Bao Yi Xue Ban 2015; 40:12–7. 10.11817/j.issn.1672-7347.2015.01.003 25652371

[11] Oliveira JM, Rebuffat SA, Gasa R, Gomis R. Targeting type 2 diabetes: lessons from a knockout model of insulin receptor substrate 2. Can J Physiol Pharmacol 2014; 92:613–20. 10.1139/cjpp-2014-0114 24977713

[12] Zhou J, Li YS, Nguyen P, Wang KC, Weiss A, Kuo YC, et al Regulation of vascular smooth muscle cell turnover by endothelial cell-secreted miR-126: role of shear stress. Circ Res 2013; 113:40–51. 10.1161/CIRCRESAHA.113.280883 23603512 PMC3772783

[13] Kubota N, Kubota T, Itoh S, Kumagai H, Kozono H, Takamoto I, et al Dynamic functional relay between insulin receptor substrate 1 and 2 in hepatic insulin signaling during fasting and feeding. Cell Metab 2008; 8:49–64. 10.1016/j.cmet.2008.05.007 18590692

[14] Dong X, Park S, Lin X, Copps K, Yi X, White MF. IRS-1 and IRS-2 signaling is essential for hepatic glucose homeostasis and systemic growth. J Clin Invest 2006; 116:101–14. 16374520 10.1172/JCI25735 PMC1319221

